# Comparison of treatment to improve gastrointestinal functions after colorectal surgery within enhanced recovery programmes: a systematic review and meta-analysis

**DOI:** 10.1038/s41598-021-86699-w

**Published:** 2021-04-01

**Authors:** Jean F. Hamel, Charles Sabbagh, Arnaud Alves, Jean M. Regimbeau, Timothée Vignaud, Aurélien Venara

**Affiliations:** 1 Faculty of Health, Department of Medicine, Angers, France; 2 Department of Biostatistics, La Maison de la Recherche, Angers University Hospital, 4 rue Larrey, 49933 Angers Cedex 9, France; 3 Department of Visceral and Endocrine Surgery, Amiens University Hospital, 1 Rond-Point du Professeur Christian Cabrol, Amiens, France; 4 Department of Visceral and Endocrine Surgery, Caen University Hospital, UMR INSERM, 1086 Anticipe, Avenue de la cote de Nacre, Caen, France; 5 Department of Visceral and Endocrine Surgery, Nantes University Hospital, 1 Place Alexis-Ricordeau, 44093 Nantes, Nantes, France; 6 Department of Visceral and Endocrine Surgery, CHU Angers, Angers University Hospital, 4 rue Larrey, 49933 Angers Cedex 9, France; 7 UMR INSERM 1235, TENS, 1 place Alexis Ricordeau, 44000 Nantes, France

**Keywords:** Gastroenterology, Gastrointestinal diseases, Functional gastrointestinal disorders

## Abstract

Despite a significant improvement with enhanced recovery programmes (ERP), gastro-intestinal (GI) functions that are impaired after colorectal resection and postoperative ileus (POI) remain a significant issue. In the literature, there is little evidence of the distinction between the treatment assessed within or outside ERP. The purpose was to evaluate the efficiency of treatments to reduce POI and improve GI function recovery within ERP. A search was performed in PubMed and Scopus on 20 September 2019. The studies were included if they compared the effect of the administration of a treatment aiming to treat or prevent POI or improve the early functional outcomes of colorectal surgery within an ERP. The main outcome measures were the occurrence of postoperative ileus, time to first flatus and time to first bowel movement. Treatments that were assessed at least three times were included in a meta-analysis. Among the analysed studies, 28 met the eligibility criteria. Six of them focused on chewing-gum and were only randomized controlled trials (RCT) and 8 of them focused on Alvimopan but none of them were RCT. The other measures were assessed in less than 3 studies over RCTs (n = 11) or retrospective studies (n = 2). In the meta-analysis, chewing gum had no significant effect on the endpoints and Alvimopan allowed a significant reduction of the occurrence of POI. Chewing-gum was not effective on GI function recovery in ERP but Alvimopan and the other measures were not sufficiently studies to draw conclusion. Randomised controlled trials are needed.

*Systematic review registration number* CRD42020167339.

## Introduction

Postoperative ileus (POI) is the slowing or stopping of gastrointestinal (GI) functions after surgery. Enhanced recovery programmes (ERP) allows a reduction in the time until GI function recovery and a decrease of the incidence of POI^1,2^. However, it still remains a time-lapse of stop of the GI functions after surgery that can lead to an impairment going from increased length of stay or pneumonia to death^3^. Also, POI has been shown to be associated with anastomotic leakage^4^.

Many treatments or mean of prevention to reduce the occurrence of POI or to reduce the time to GI function recovery has been assessed and analysed through meta-analyses but the interpretation of such literature is difficult due to the lack of identification of the perioperative management within the different studies and meta-analyses whilst it can be a serious bias.

ERP first appeared in Scandinavia in the 2000s before being generalised and it is difficult to identify studies in which the patients have been treated using ERP, a conventional treatment or both. It is important to know this because it can influence the results and some items that work with conventional treatment do not work with ERP. For example, it has been shown that chewing gum was efficient in the conventional management literature^5^, but not in ERP^6^. To date, there is no systematic review that draws a picture of the therapeutics or prophylactics that have been assessed in literature within ERP. Such a review could help physicians in the management of their patients, in improving and simplifying ERP, and such a review could help researchers to have a better cartography of “what works, what does not work and what has been assessed”.

The main aim of this study is to provide a systematic review of literature to draw a picture of the different means that were used to prevent or to treat POI and to improve GI function recovery. The second aim of this study is to provide a meta-analysis and a network meta-analysis of all the previously described means of treatment or prevention in order to offer the strongest proof of the efficiency, or lack thereof, of such means.

## Methods

The study was registered at PROSPERO (registration number CRD42020167339) and it was reported according to PRISMA guidelines.

The inclusion criteria were: studies comparing at least two treatments that are not a part of the ERP that could improve the time to the first flatus or the first bowel movement, or reduce the occurrence of POI after colorectal surgery during the ERP. Studies were excluded if they were reviews or meta-analyses, case reports, letters, study protocols, on children, on animals or in a language other than French or English. Studies were also excluded if the material and methods did not specify if the setting was an ERP or not, or if such section specified that the setting was not an ERP. Studies were not included in the meta-analysis if the intervention was a feature used in the ERP (such as multimodal analysis, early feeding…).

The first outcome measure was POI occurrence. The definition of POI was recorded for all the studies included in the meta-analysis. POI could be the first outcome measure or the secondary outcome measure as well as it was reported in the study.

Secondary outcome measures were the time to first flatus and the time to first bowel movement.

A systematic search of the PubMed and Scopus databases was performed until 20 September 2019. The search algorithm in PubMed was as follow: ("2000/01/01"[Date–Publication]: "3000"[Date–Publication]) AND (("colon"[Title/Abstract]) OR ("rectal"[Title/Abstract]) OR ("colorectal"[Title/Abstract])) AND (("surgery"[Title/Abstract]) OR ("postoperative"[Title/Abstract])) AND (("ileus"[Title/Abstract]) OR ("motility"[Title/Abstract])) (Table 1).

**Table 1:** Search strategy in the databases.

"2000/01/01"[Date–Publication]: "3000"[Date–Publication]	AND	("colon"[Title/Abstract])	AND	("surgery"[Title/Abstract])
		OR		OR
		("rectal"[Title/Abstract])		("postoperative"[Title/Abstract])
		OR		OR
		("colorectal"[Title/Abstract])		(("ileus"[Title/Abstract])
				OR
				("motility"[Title/Abstract])

All the titles and abstracts were extracted into an Excel sheet. Duplicates were removed. The titles and abstracts were reviewed by the authors to identify those that met the inclusion criteria. Full text screening was conducted by two authors independently. The data extraction was also conducted by two independent authors.

When the time to first flatus or bowel movement was reported as a median and interquartile range (IQR) or as a mean and standard error of the mean (SEM) in the original articles, these values were transformed as mean and standard deviation by assuming a normal distribution, so as to allow them to be included in the network meta-analysis.

Risk of bias were assessed by two authors, using the ROB-2 tool when studying randomised controlled trials (RCTs) and the ROBINS-I tool when studying non-randomized studies. Consensus was performed with a third author, if necessary.

### Meta-analysis

We conducted meta-analyses based on random-effects models using the Knapp-Hartung method and the Sidik-Jonkman estimator^7–9^. Direct and indirect evidence for all studied treatments were combined to evaluate their associations with the studied outcomes. Results were then presented as odds ratios when studying dichotomous endpoints like ileus, and as standardised mean difference when studying continuous endpoints like time to first flatus or bowel movement.

These pairwise meta-analyses were only conducted if at least three studies assessed the same process.

Statistical heterogeneity was quantified with the I^2^ and τ^2^ statistics and tested using the Q-test of heterogeneity. When a significant heterogeneity was highlighted, meta-analyses were carried out again, excluding outlier studies, to evaluate the consistency of the meta-analysis results. Publication bias was assessed by the Egger regression asymmetry test.

All analyses were done using R software with the Meta, Netmeta and Dmetar packages (*R software, 3.6.3, *https://www.R-project.org/). All the statistical tests were bilateral considering a p-value threshold set at 0.05.

## Results

### Literature search

**Figure 1:**
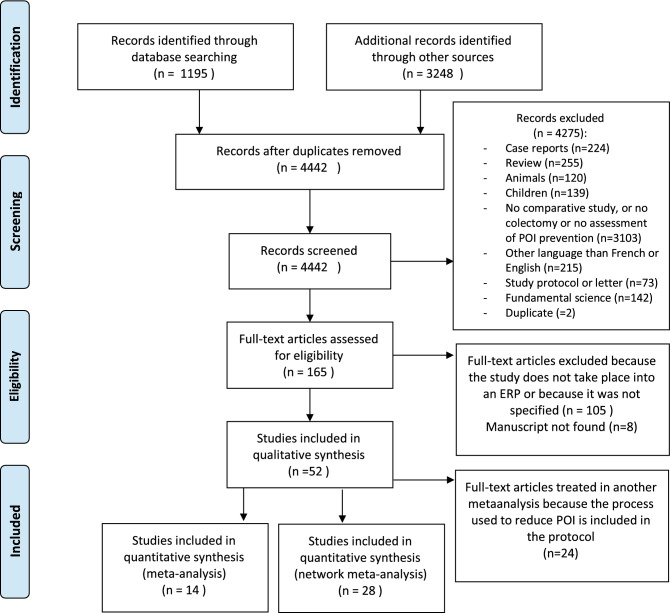
PRISMA flow chart.

We identified 4442 articles after removing the duplicates, 4275 were excluded by screening the abstract because they did not meet the inclusion criteria and 167 full texts were analysed in their whole form (Fig. 1). Among these articles, 115 were excluded because the manuscript was not available or because the study did not take place within an ERP. Of the 52 remaining articles on studies, 24 concerned the ERP: analgesia or anaesthesia management for 17 studies^10–23^, exercise therapy for 1^24^, type of anastomosis for 2^25,26^, diet for 4^27–30^ and the need for carbohydrate-rich beverages for 1^31^. As these studies were concerned with different elements of ERP and not with specific supplementary treatments for GI function recovery, they were not included in the meta-analysis.

### Narrative synthesis

The other 28 processes are treatments or prophylaxis for POI or treatments to improve GI function recovery, such as alvimopan (n = 8)^32–39^, chewing gum (n = 6)^40–45^, coffee intake (n = 3)^46–48^, the use of prokinetic drugs (n = 5) such as ghrelin^49,50^, magnesium^51^, prucalopride ^52^, simethicone syrup ^53^; the use of non-steroidal anti-inflammatory drugs (NSAIDs) (n = 2)^54,55^, the use of furosemide (n = 1)^56^, the use of simvastatin (n = 1)^57^, the use of Gastrografin (n = 1)^58^ and the use of transcutaneous tibial nerve stimulation (TTNS) (n = 1)^59^. Of these, 18 were randomised controlled trials. The design of the studies is reported in Table 2.

**Table 2:** Systematic review of the studies assessing preventive treatment for POI.

First author	Design	Inclusions (n =)	Surgery performed	Outcomes assessed	Definition of POI	Reference treatment	Experimental treatment
Lim et al.^44^	RCT	161	L or O colorectal resection	FF, FBM		N	CG
Zaghyian et al.^43^	RCT	114	L or O major colorectal surgery	FF, FBM, POI	Postoperative nausea/vomiting, accompanied by abdominal distension, absence of bowel function and X-ray findings consistent with POI	N	CG
Byrne et al.^42^	RCT	158	L or O bowel surgery	FF, FBM, POI	ND	N	CG
Shum et al.^40^	RCT	86	L colorectal resection	FF, FBM		N	CG
Yang et al.^41^	RCT	565	L or O bowel resection	FF, FBM		N	CG^a^
Atkinson et al.^45^	RCT	412	L or O colorectal resection	FF, FBM, POI	ND	N	CG
Itawi et al.^35^	R	165	L colectomy	POI	Delay of return of bowel function ≥ 36–48 h	N	A
Ludwig et al.^34^	Post-hoc	1409	O bowel resection	POI		P	A
Barletta et al.^37^	R	282	L or O colectomy	POI	3 episodes of vomiting over 24 h, cessation of oral diet, and need for NGT within 5 PODs	N	A
Obokhare et al.^33^	R	200	Laparoscopic colectomy	POI	Lack of recovery of GI function within 3 POD, or insertion of an NGT	N	A
Wen et al.^38^	R	116	L or O colorectal resection	POI	Abdominal distension, failure to pass flatus or stool and nausea and emesis with placement of an NGT	N	A
Adam et al.^36^	P	660	L or O colorectal resection	POI	Reinsertion of NGT	N	A
Hyde et al.^32^	R	636	L and O colorectal resection and ostomy reversal	POI	Absence of GI motility recovery ≥ 5 days or need for NGT	N	A
Keller et al.^39^	R	642	L colorectal resection	POI	ND	N	A
Müller et al.^46^	RCT	79	Colonic surgery	FF, FBM		HW	C
Hasler-Gehrer et al.^47^	RCT	115	L and O colonic resection	FF, FBM		T	C
Dulskas et al.^48^	RCT	105	Laparoscopic left-sided colectomy	FF, FBM		HW	C^b^
Springer et al.^53^	RCT	118	L or O colorectal resection	FF, FBM, POI	ND	P	S
Danelich et al.^56^	RCT	123	L or O colon and rectal surgery	FBM, POI	ND	N	F
Andersen et al.^51^	RCT	49	Open elective surgery	FF, FBM		P	Mg
Raju et al.^55^	R	252	Major abdominal surgery	POI	Return to fasting, reinstitution of IV, cessation of GI functions for ≥ 5 days, parenteral nutrition	Retrospective cohort	NSAID
Lohsiriwat et al.^54^	R	150	L colorectal resection	FBM, POI	Vather's definition	N	NSAID
Singh et al.^57^	RCT	132	L and O colorectal resection	FF, FBM, POI	Nausea or vomiting with inability to tolerate oral intake and requiring the insertion of NGT	P	Statine
Gong et al.^52^	RCT	110	L and O GI surgery	FF, FBM, POI	Vather's definition	P	Pr
Popescu et al.^49^	RCT	236	O partial colectomy	FF, POI	ND	P	G TZP-101 80 mg^c^
Falken et al.^50^	RCT	24	Colorectal surgery	FF		P	G
Venara et al.^59^	RCT	40	O and L colorectal resection	FF, POI	Absence of GI motility recovery > 4	P	TTNS

MA: meta-analysis, NMA: network meta-analysis, RCT: randomised controlled trial, R: retrospective, FF: time to first flatus, FBM: time to first bowel movement, POI: postoperative ileus, L: laparoscopic, O: open, GI: gastrointestinal, ND: not defined, POD: postoperative day, NGT: nasogastric tube, P: placebo, N: none, P: placebo, CG: chewing gum, A: alvimopan, C: Coffee, S: simethicone syrup, F: Furosemide, NSAID: non-steroidal anti-inflammatory drugs, Pr: prucalopride, G: ghrelin, TTNS: transcutaneous tibial nerve stimulation.

Third group: ^a^Acupuncture, ^b^Decaffeinated, ^c^G TZP-101 480 mg.

Only one study assessed a curative treatment for POI. Indeed, in one RCT including 29 patients, the authors assessed the reduction of the time to resolve POI for patients receiving gastrografin as compared to a placebo^58^. No significant difference was shown between the placebo group (10.3 days (CI95% 6.96–10.29) and the Gastrografin group (9.1 CI95% 6.51–11.68) (p = 0.878).

The other 27 studies assessed treatment to reduce the time to GI function recovery to prevent POI (Table 3).

**Table 3:** Systematic review of the results of the studies assessing preventive treatment for POI.

First author	Unity	Time to FF (ref)	Time to FF (exp)	Time to FBM (ref)	Time to FBM (exp)	POI (%) (ref)	POI (%) (exp)
Lim et al.^44^		50.97 ± 3.79	42.75 ± 3.92	98.61 ± 7.06	89.64 ± 5.94	–	–
Zaghyian et al.^43^	Hours	47.4 (29.4)	48.6 (33.4)	63.2 (41.9)	56.9 (37.8)	6.6%	7.4%
Byrne et al.^42^	Hours	58.0 (42.0–74.0	42.0 (36.4–47.6)	**80.0, 67.2–92.8**	**50.0, 45.2–54.8**	17%	10%
Shum et al.^40^	Hours	**34 (7–144)**	**18 (5–90)**	**44 (9–152)**	**19 (5–81)**	–	–
Yang et al.^41^	Hours	64.1 (24.8–71.3)	62.3 (21.4–70.5) (CG)	75.2 (29.0–241.6)	119.3 (31.5–211.4) CG)	–	–
Atkinson et al.^45^	Days	2 (1–3)	2 (2–3)	3 (1–4)	2 (1–3)	14.1%	9.4%
Itawi et al.^35^		–	–	–	–	**20%**	**2%**
Ludwig et al.^34^		–	–	–	–	-	-
Barletta et al.^37^		–	–	–	–	21.7%	16.4%
Obokhare et al.^33^		–	–	–	–	**12%**	**4%**
Wen et al.^38^		–	–	–	–	10.3%	3.4%
Adam et al.^36^		–	–	–	–	**16%**	**5%**
Hyde et al.^32^		–	–	–	–	**16.2%**	**10.8%**
Keller et al.^39^		–	–	–	–	2.2%	2.2%
Müller et al.^46^	Hours	46.4 (20.1)	40.6 (16.1)	**74.0 (21.6)**	**60.4 (21.3)**	–	–
Hasler-Gehrer et al.^47^	Hours	31 (25–46)	40 (29–52)	**74.1 (60.7–87.5)**	**65.2 (50.5–79.8)**	–	–
Dulskas et al.^48^	Days	**1.57**	**1.77 (Coffee)**	**4.14 (± 1.15)**	**3.75 (± 1.53)**	–	–
Springer et al.^53^	Hours	37.9 ± 23.9	37.6 ± 26.7	41.1 ± 28.2	42.1 ± 25.2	4.7%	3.5%
Danelich et al.^56^		–	–	**31.8 (22.5–54.4)**	**52.9 (45.3–82.4)**	15.7%	20.5%
Andersen et al.^51^		14.0 (6–46)	18.0 (6–62)	50.0 (6–70)	42.0 (14–110)	–	–
Raju et al.^55^		–	–	–	–	**13.4%**	**7.23%**
Lohsiriwat et al.^54^		–	–	**3 (3–4)**	**2 (2–3)**	8%	5%
Singh et al.^57^		1 (1–3)	2 (1–2)	4 (1–5)	4 (2–7)	22%	18%
Gong et al.^52^		**73.0 (7–305)**	**51.0 (26–129)**	**94.5 (27–315)**	**64.0 (25–172)**	35.7%	17.8%
Popescu et al.^49^		**2.85** **_2.31, 2.93_**	**2.20** **_1.92, 2.80_**	–	–	0	4.2%
Falken et al.^50^	days	**3.5 (± 0.4)**	**2.1 (± 3)**	–	–	–	–
Venara et al.^59^	days	2.16 ± 0.32	1.47 ± 0.19	–	–	**42%**	**6.1%**

Bold indicate significant difference.

FF: time to first flatus, FBM: time to first bowel motion, POI: postoperative ileus, Exp: experimental, Ref : reference, N: none, P: placebo, CG: chewing gum.

The Alvimopan was the most studied preventive treatment of POI but it was only assessed through non RCT studies^35,37,39–41^. Those studies exclusively compared the rate of POI and 4 of them showed a significant reduction of the rate of POI in the patients taking Alvimopan (2–10.8%) as compared to the patients not taking Alvimopan (12–20%)^32,33,35,36^. Ludwig et al.^34^ assessed the efficiency of Alvimpan to reduce the time to GI2 recovery. They showed a non-significant difference (21% vs 34%).

The chewing gum has been assessed in 6 RCTs^42–47^. Three of those compared the rate of POI but none reported significant difference between chewing gum (7.4–10%) and the control group (6.6–17%)^42,43,45^. Finally, only 1 of those 6 studies reported a significant reduction of the time to first flatus^40^ and 2 of those studies reported a significant reduction of the time to first bowel motion^40,42^. The study from Yang et al.^41^ compared 3 arms of treatments (control, chewing-gum, simo-decoction + acupuncture). They reported a higher improvement of GI function by using simo decoction + acupuncture as compared to chewing-gum or as compared to the control group.

Three studies assessed the coffee to reduce the time to first flatus or first bowel motion. Two of them compared the coffee to hot water^46,48^ and one of them to tea^47^. The control group being different, no meta-analysis was performed. All those studies showed a reduction of the time to first bowel motion^46–48^ and only one showed a reduction of the time to first flatus^48^. This last study also interested in decaffeinated coffee. This coffee without caffeine reduced the time to first flatus and first bowel motion as compared to the control group and to coffee with caffeine.

The intake of furosemide significantly increased the time to first bowel motion in one RCT^56^ while NSAIDs significantly reduced the time to first bowel motion in one retrospective study^54^ and significantly reduced the rate of POI in one retrospective study^55^.

The prucalopride and the ghrelin showed a significant of the time to first flatus in 3 RCTs^49,50,52^. Finally, in per-protocol analysis, TTNS significantly reduced the rate of POI in one RCT^59^.

### Risk of bias

**Figure 2:**
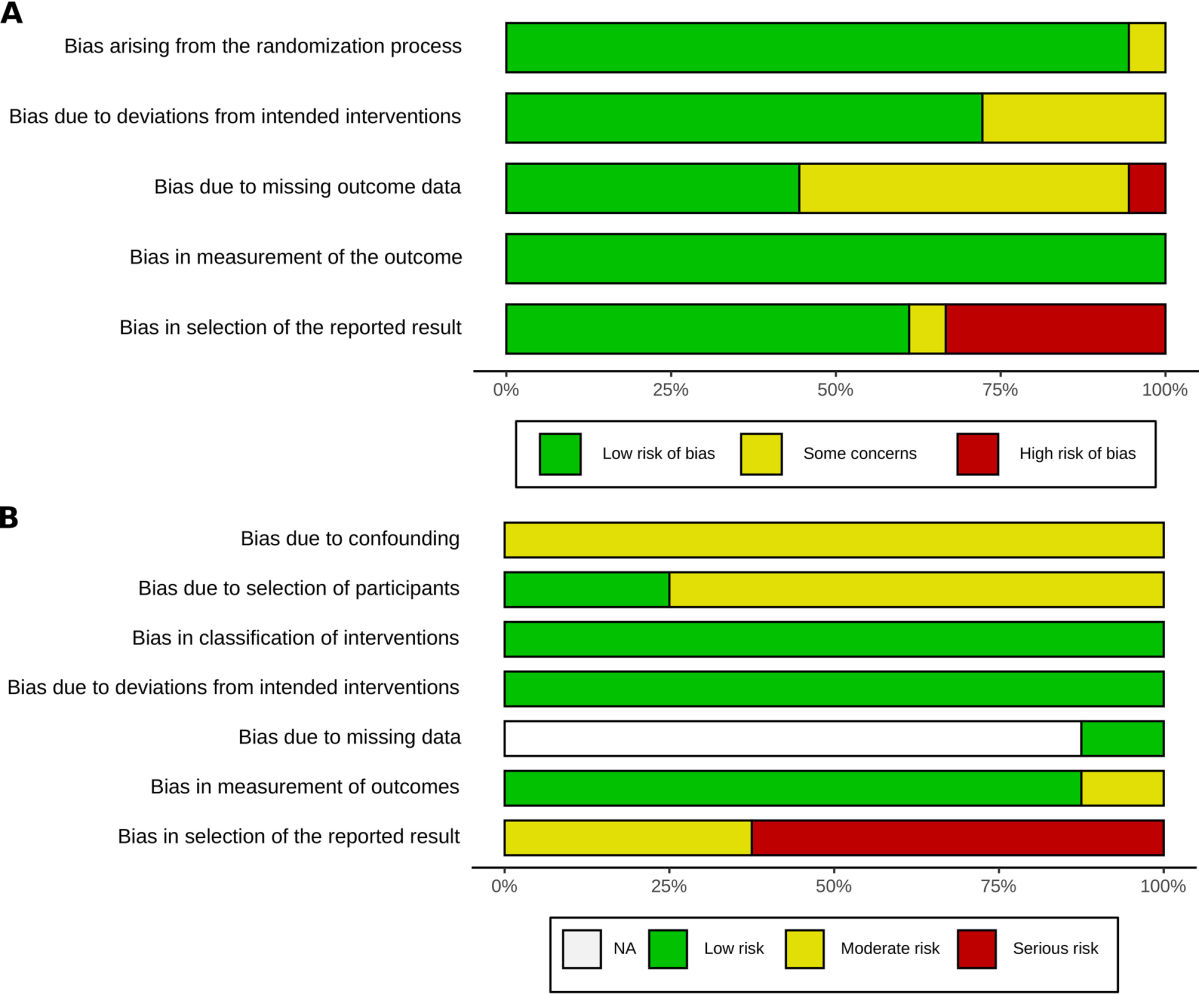
(**A**) Bias assessement bias assesment tool for RCT's (ROB-2) and (**B**) bias assesment tool for non randomized studies (ROBINS'I).

Several studies were identified as presenting serious risks of biases, considering both randomized and non-randomized studies. Two factors explained the vast majority of them: the missing data management and the lack of identification of a single primary objective (Table 4, Fig. 2). The outcome assessment blinding process was not informed in many studies. Many studies did not mention the missing data rate. For those mentioning such a missing data rate, five studies presented a lost-to-follow-up rate greater than 10%. Two studies reported commercial funding for the study even if the data analysis was reported to be independent.

**Table 4:** Assessment of the risk of bias.

Article	Randomization process	Deviations from intended interventions	Missing outcome data	Measurement of the outcome	Reported results		
Lim et al.^44^	Low	Low	Some concerns	Low	Low		
Zaghyian et al.^43^	Low	Low	Low	Low	Low		
Byrne et al.^42^	Some concerns	Low	Some concerns	Low	High		
Shum et al.^40^	Low	Low	Some concerns	Low	High		
Yang et al.^41^	Low	Low	Low	Low	High		
Atkinson et al.^45^	Low	Low	Some concerns	Low	Low		
Müller et al.^46^	Low	Low	Low	Low	Low		
Hasler-Gehrer et al.^47^	Low	Some concerns	Low	Low	Low		
Dulskas et al.^48^	Low	Some concerns	Some concerns	Low	Some concerns		
Springer et al.^53^	Low	Low	Low	Low	Low		
Danelich et al.^58^	Low	Low	Low	Low	Low		
Andersen et al.^56^	Low	Some concerns	High	Low	High		
Singh et al.^57^	Low	Low	Some concerns	Low	Low		
Gong et al.^52^	Low	Low	Low	Low	Low		
Popescu et al.^49^	Low	Some concerns	Low	Low	Low		
Falken et al.^50^	Low	Low	Some concerns	Low	High		
Venara et al.^59^	Low	Some concerns	Some concerns	Low	Low		

	Confounding	Selection of participants	Classification of interventions	Deviations from intended interventions	Missing data	Measurement of outcomes	Reported results
Itawi et al.^35^	Moderate	Low	Low	Low	No information	Moderate	Serious
Barletta et al.^37^	Moderate	Moderate	Low	Low	No information	Low	Serious
Obokhare et al.^33^	Moderate	Moderate	Low	Low	No information	Low	Serious
Wen et al.^38^	Moderate	Moderate	Low	Low	Low	Low	Moderate
Adam et al.^36^	Moderate	Moderate	Low	Low	No information	Low	Moderate
Hyde et al.^32^	Moderate	Moderate	Low	Low	No information	Low	Moderate
Keller et al.^39^	Moderate	Moderate	Low	Low	No information	Low	Serious
Lohsiriwat et al.^54^	Moderate	Low	Low	Low	No information	Low	Serious

### Meta-analysis

#### Alvimopan

**Figure 3:**
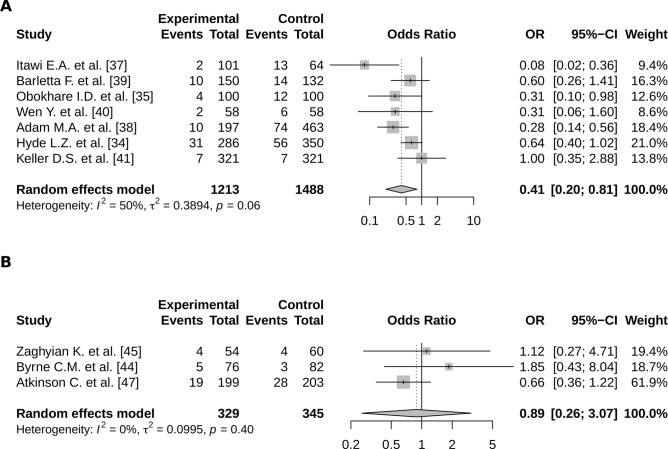
Meta-analysis of the risk of POI according to a treatment by (**A**) alvimopan and (**B**) chewing gum; (R software, 3.6.3, https://www.R-project.org/).

Among the eight studies focusing on alvimopan, seven assessed its impact on POI and none assessed the impact on time to first flatus or to first bowel movement (Table 1). The pairwise meta-analysis highlighted a significant reduction of POI occurrence (OR = 0.41; CI95% 0.20–0.81) (Fig. 3A).

#### Chewing gum

Six RCTs studied the impact of chewing-gum on POI^40–44^. These six studies included both first flatus and first bowel movement as outcome measures. Only three included POI as an outcome measure (Table 1).

No significant effect of chewing gum was highlighted concerning the POI occurrence, the time to first flatus, nor the time to first bowel movement (respectively OR = 0.89; CI95% 0.26–3.07, SMD = − 0.07; CI95% − 0.19;0.06 and SMD = − 0.24; CI95% − 0.50–0.01) (Fig. 3B).

**Figure 4:**
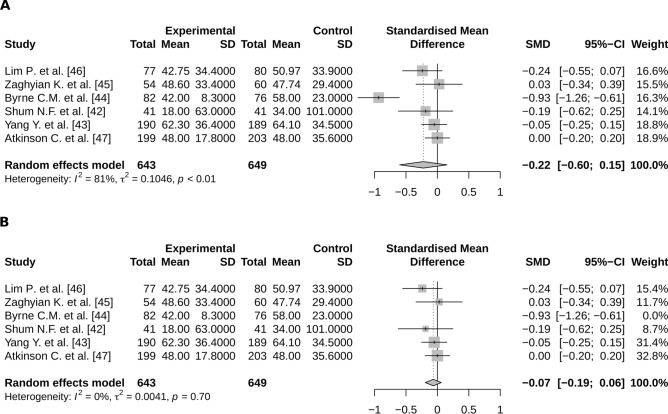
Meta-analysis of the time to first flatus according to a treatment by chewing gum (**A**) with and (**B**) without the outlier; (R software, 3.6.3, https://www.R-project.org/).

**Figure 5:**
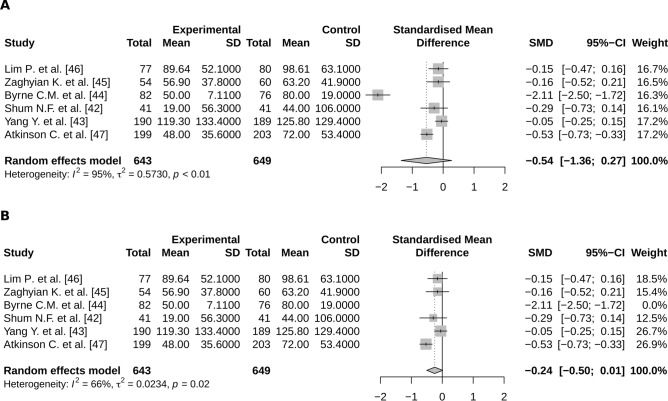
Meta-analysis of the time to first bowel movement according to a treatment by chewing gum (**A**) with and (**B**) without the outlier (R software, 3.6.3, https://www.R-project.org/).

Significant heterogeneity was observed concerning studies focusing on the relationship between chewing gum and time to first flatus (Fig. 4A) or to first bowel movement (Fig. 5A). When excluding the outlier studies from these meta analyses, the effect of chewing gum on both time to first flatus and time to first bowel movement interestingly became closely significant (respectively SMD = − 0.07; 95%CI − 0.19:0.06 and SMD = − 0.24; 95%CI − 0.5;0.01) (Figs. 4B and 5B).

## Discussion

Among the analysed studies, 28 were concerned with the effect of different measures on preventing POI or reducing GI function recovery. 12 drugs were assessed but only two were assessed at least three times. The Alvimopan was the most studied preventive treatment of POI through non RCT studies. Four of them showed a significant reduction of the rate of POI in the patients taking Alvimopan. The chewing gum has been assessed in 6 RCTs. None of them reported a significant improvement in the chewing-gum group, one reported a significant reduction of the time to first flatus and 2 reported a significant reduction of the time to first bowel motion. Also, 3 studies assessed the coffee and showed a reduction of the time to first bowel motion and only one showed a reduction of the time to first flatus.

In the meta-analysis including six RCTs, chewing gum had no significant effect on the endpoint but was close to reducing the time to first flatus and first bowel movement. In the meta-analysis including seven non-RCTs, alvimopan allowed a significant reduction of the occurrence of POI.

Interestingly, many studies assessed the impact of chewing gum on GI functions before the ERP. These studies largely showed a significant reduction of the time to GI function recovery or of the occurrence of POI^5,60–64^. Some authors support the opinion that chewing gum is not cost effective in the ERP because patients are allowed to drink and eat at an early stage, leading to a natural vagal stimulation that reduces the duration of GI function impairment^64^. However, all these studies assessed chewing gum with a view to preventing POI or reducing GI function recovery, but none assessed the utility of sham feeding in patients already presenting nausea or vomiting or requiring a nasogastric tube. This may be a new way for using chewing gum, not as a prevention but as a treatment for POI. This may improve postoperative care when we know that early feeding tolerance has been shown to be a predictive factor of the outcomes of colorectal surgery.

Then, alvimopan showed considerable utility by reducing POI. Many meta-analyses performed using studies that took place during, or outside, the ERP showed a reduction of the occurrence of POI by using such a drug^65,66^. The fact that alvimopan was efficient on POI even within ERP is surprising because it inhibits the peripheric opioid receptors while, theoretically, the patients should not receive high doses of morphine in ERP^67^. This result should therefore be considered with caution because the studies included in the meta-analysis were not RCTs and had some bias, such as the POI definition that was different between the studies. Therefore, the effect of alvimopan could be overestimated. Despite these encouraging results, a well-designed RCT is probably needed to confirm this result.

Coffee was assessed in three RCTs and showed a significant effect on the reduction of the duration of GI function impairment. This is interesting while it was shown that it also reduced these outcomes outside the ERP^68^. The caffeine was supposed to reduce inflammation in the bowel by stimulating the vagal pathway^69^.

The narrative review showed a potentially beneficial effect of NSAIDs on the first bowel movement. This is possible because it could be explained by the physiopathology of the POI, and the involvement of the inflammation in the installation of such pathology^70,71^. This must, however, be confirmed with RCTs to improve the quality of proof.

Also, the intake of tea or ghrelin and acupuncture possibly improved the time to first flatus. Again, this could be explained by the physiopathology for the ghrelin. Indeed, ghrelin stimulates GI motility and contributes to energy homeostasis^72^. We have no explanation for why tea or acupuncture could improve GI function recovery, but the level of proof is very low in this case (only one study for each modality of treatment, with three arms for each study).

Despite these encouraging results, two major difficulties in the analysis of POI within ERP have to be raised. First of all, the definition of POI was significantly different between the studies or was not reported in the material and methods section. This lack of consensual definition has already been raised in literature in 2005 by Kelhet et al.^73^ and still existed in 2017 despite multiple effort from physician to better understand and define POI^74^. This lack of consensual definition is a common limitation in all the review on POI management or prevention. Unfortunately, the study of bowel function recovery cannot supply to the lack of definition of POI because the outcome measures for bowel function recovery is not much consensual and vary between studies^75^, leading to discrepancy between studies. A study is now in progress to improve this issue but the results are not yet available^76^.

The second issue is due to the heterogeneity of ERP between the different teams and countries. Some of the studies did not describe the ERP while the others reported different items. This bias has been raised in 2015^77^ and is still an issue in current literature. Indeed, to improve the quality of studies reporting ERP management, the ERAS Society proposed a checklist containing 20 items to better assess ERP compliance^78^. This will probably homogenize the studies on the influence of ERP on diverse pathology such as POI. In this meta-analysis, only studies reporting the patients followed an ERP were included in order to homogenize the perioperative management of the patient and to specifically focus on the treatment assessed.

Finally, the treatment that seemed to be more efficient was only assessed in non-RCTs. This could represent a bias of recruitment, but also in the reproducibility of the study because the definition was different between the studies and could be different within a single study.

These limitations preclude a reliable metaanalysis. Unfortunately, until there is no consensual definition of POI and until the ERP is not consensually reported, it will be difficult to bring stronger conclusion on all those elements used in addition ERP. This narrative review and meta-analysis brings some evidence on the efficiency of such elements. The aim of this meta-analysis was therefore achieved because (i) it raised the difficulties on assessing the treatments of POI within ERP and (ii) it structured the different modalities of treatment. Further RCTs are therefore needed to confirm whether there are beneficial effects of such treatment.

## Conclusion

This meta-analysis revealed that, in ERP, the improvement of GI function recovery by measures, and especially the POI, is poorly studied in literature, with high discrepancy on definitions of POI and ERP. No strong conclusions can be drawn, except that chewing gum and coffee had no beneficial effect on these endpoints. Alvimopan reduced the occurrence of POI but further RCTs are needed to confirm this effect.
